# Adult Neurogenesis and Glial Oncogenesis: When the Process Fails

**DOI:** 10.1155/2014/438639

**Published:** 2014-03-11

**Authors:** Chary Marquez Batista, Eric Domingos Mariano, Breno José Alencar Pires Barbosa, Matthias Morgalla, Suely Kazue Nagahashi Marie, Manoel Jacobsen Teixeira, Guilherme Lepski

**Affiliations:** ^1^Department of Neurology, School of Medicine, University of São Paulo, Avenida Dr. Arnaldo 455, LIM 15, 4th Floor, 01246-903 Cerqueira Cesar, SP, Brazil; ^2^Center for Cellular and Molecular Studies and Therapy-NAP-NETCEM, University of São Paulo, Brazil; ^3^Department of Neurosurgery, Eberhard Karls University, Tuebingen, Germany

## Abstract

Malignant brain tumors, including glioblastoma multiforme (GBM), are known for their high degree of invasiveness, aggressiveness, and lethality. These tumors are made up of heterogeneous cell populations and only a small part of these cells (known as cancer stem cells) is responsible for the initiation and recurrence of the tumor. The biology of cancer stem cells and their role in brain tumor growth and therapeutic resistance has been extensively investigated. Recent work suggests that glial tumors arise from neural stem cells that undergo a defective process of differentiation. The understanding of this process might permit the development of novel treatment strategies targeting cancer stem cells. In the present review, we address the mechanisms underlying glial tumor formation, paying special attention to cancer stem cells and the role of the microenvironment in preserving them and promoting tumor growth. Recent advancements in cancer stem cell biology, especially regarding tumor initiation and resistance to chemo- or radiotherapy, have led to the development of novel treatment strategies that focus on the niche of the stem cells that make up the tumor. Encouraging results from preclinical studies predict that these findings will be translated into the clinical field in the near future.

## 1. Introduction

Glioblastomas account for the great majority of primary brain tumors in adults. Despite multimodality treatments, the prognosis remains poor, with a median survival time of approximately 1 year following the diagnosis of glioblastoma [1–4]. How can such an aggressive tumor arise in the brain, a carefully orchestrated organ, where cellular proliferation is barely needed to maintain function? Over the past two decades, genetic, cell biological, and animal modeling studies have led to a better understanding of the formation and progression of malignant glioblastomas. The origin of these tumors, however, is not fully understood.

While early data suggested that glioblastomas originate from normal glial cells, more recent data suggest they may in fact arise from neural stem cells or neural progenitors [5, 6]. The cancer stem cell (CSC) hypothesis suggests that neoplastic clones are maintained exclusively by a rare fraction of cells with stemness properties [5]. Glioblastomas contain multipotent tumor stem cells that could be responsible for populating and repopulating tumors [7].

Even though there is no evidence showing that most brain cells undergo division during adult life, the idea of a “window of neoplastic vulnerability” implies that oncogenic events may occur in still-proliferating fetal cells [7]. According to this theory, since neuronal cells divide (and undergo oncogenic events) early during embryogenesis, neuronal tumors such as medulloblastomas occur mostly early in life. Glial tumors, however, are more common and arise later in life, because glial proliferation occurs later.

The existence of CSCs has major therapeutic implications. These cells have been isolated and characterized as a heterogeneous population with unique features, giving them a key status in tumor survival. From a therapeutic standpoint, a critical issue is to identify and understand the physiology of the cell(s) responsible for tumor formation and recurrence. Therapies that do not ablate the tumor stem cells will be ineffective in eradicating the tumor. These stem cells may be transformed variants of normal neural progenitor cells, but the functional identity of these cells (i.e., stem cells or neural progenitor cells) remains controversial [4, 6, 7].

The present review aims to describe the role of CSCs in the initiation and development of glioblastomas, as well as their involvement in therapy resistance. To this end, we first address the mechanisms beyond normal adult neurogenesis, and secondly, the biochemical and genetic processes that drive cells towards tumor formation.

## 2. Adult Neurogenesis

Stem cells are immature cells with the capacity for self-renewal and differentiation. Multipotent neural stem cells (NSCs) have the ability to differentiate into neurons and glia (astrocytes and oligodendrocytes) [35–37]. The process of neurogenesis, which consists in the formation of new neurons from neural stem/progenitor cells, occurs in two major regions of the adult mammalian brain: in the subventricular zone of the lateral ventricles (SVZ) and in the subgranular layer of the hippocampal dentate gyrus (SGZ). In the adult central nervous system (CNS), these new neurons are integrated into the mature neuronal circuitry and take on various functions, thereby contributing to the structural and functional plasticity of the system [38, 39].

### 2.1. Subventricular Zone

The subventricular zone is the largest neurogenic region of the adult brain. In this region, the true physiological NSCs are a special type of astrocyte positive for glial fibrillary acidic protein (GFAP) and known as type B cells. These astrocytes divide asymmetrically at a low duplication rate, producing a cell resembling itself and another small rounded cell (i.e., type C cells). These type C cells duplicate at a high rate and are therefore called transit-amplifying cells (TACs). These rapidly dividing TACs produce neuroblasts or neural progenitors that form aggregate chains which migrate at high speeds from the SVZ toward the olfactory bulb (OB) through the rostral migratory stream (RMS). Thereafter, these immature neurons differentiate mostly into granule neurons and a small proportion of them become periglomerular neurons. These two types of neurons are GABAergic, are functionally integrated into mature circuits of OB, and are constantly replaced throughout life [40, 41].

### 2.2. Subgranular Zone in the Dentate Gyrus

Similarly to what occurs in the SVZ, granule neurons arise from NPCs in the subgranular zone of the hippocampal dentate gyrus. The NSCs of this region are also a subset of special astrocytes [42] that populate the border between the hilus and the granule cell layer [43]. When activated, these types of B cells give rise to TACs; after a limited number of cell divisions, these TACs generate neuroblasts (or immature neurons) and are committed to a particular neuronal lineage. The maturation of these cells generates granular neurons, which are then integrated into preexisting hippocampal circuits. These new granule neurons extend their axons toward the molecular layer, receive afferents from the entorhinal cortex, and project their axons (called mossy fibers) toward the CA3 region, synapsing with CA3 interneurons and pyramidal cells. These mossy fibers exhibit glutamatergic terminals, indicating the formation of excitatory synapses [44].

### 2.3. Regulation of Adult Neurogenesis

NSCs are regulated by the integration of intrinsic factors with extrinsic signals from the surrounding microenvironment, known as neurogenic niche. A niche can be defined as the limited and specialized anatomic compartment formed by cellular and acellular components that integrates local and systemic factors, supports maintenance and survival, and actively regulates the function and proliferation of these cells [45].

The process of neurogenesis depends on a complex cascade of molecular signaling pathways. The candidate pathways for regulating neuronal differentiation of adult NSCs include Notch [46], bone morphogenetic protein (BMP) [47], Wnt [38], and sonic hedgehog (Shh) [48].

Neurotrophic factors also play an important role in adult neurogenesis, as they can regulate various stages of neuronal development, including their complete maturation. Brain-derived neurotrophic factor (BDNF) and neurotrophin-3 (NT-3) are considered powerful molecular mediators in synaptic and morphological plasticity [49]. BDNF can induce proliferation, survival, and neuronal differentiation, most likely by inducing the expressions of Na^+^ and K^+^ channels and the synaptic maturation of NPCs [50–52]. NT-3 has also been shown to influence neuronal survival, proliferation, and differentiation [53, 54]. Other neurotrophic and growth factors have also been shown to regulate NSCs, for example, fibroblast growth factor 2 (FGF-2), epidermal growth factor (EGF), transforming growth factor (TGF), ciliary neurotrophic factor (CNTF), and vascular endothelial growth factor (VEGF). Studies in which these molecules were administered have reported an increase in cellular survival and proliferation rates [55].

## 3. Gliogenesis

As discussed above, adult neurogenesis triggers remodeling of the neuronal circuitry through the addition of new neurons; however, it has also been shown that when deregulated, NSCs and their progenitors can lead to the formation of certain types of brain tumors, including glioblastoma multiforme (GBM).

Brain tumors are composed of different cell populations differing in phenotype and functional features. Most of the cells that make up the tumor mass appear to be nontumorigenic, and only a small subpopulation of cells (i.e., cancer stem cells (CSCs)) is responsible for tumor initiation and recurrence [56]. The presence of CSCs in brain tumors was first reported following the isolation of clonogenic stem cell-like spheres from human GBM tissue [57].

There are several theories regarding the origin of CSCs. One hypothesis is based on the idea that CSCs are derived from physiological stem cells that acquire the ability to generate tumors following genetic mutations or environmental alterations. This can occur because physiological stem cells have a long life expectancy and divide frequently, which makes them more susceptible to becoming tumorigenic [58]. The B type cells of the SVZ and SGZ are normally in a quiescent state and proliferate rapidly when necessary. One of the stages that is most susceptible to cell transformation is the transition of NSCs into TACs, because it involves a rearrangement in chromatin and rapid proliferation. Thus, if a genetic lesion is not fixed and remains within that cell, it becomes incorporated into the dividing cells, increasing the risk of other injuries and, consequently, giving rise to a cancerous cell [4, 59].

Glioma stem-like cells (GSCs) have many properties similar to those of NSCs, such as the capacity for self-renewal, proliferation, migration, and differentiation into at least one specific lineage. Also, they express common sets of markers and share signaling pathways responsible for proliferation [38, 56].

CD133 is a transmembrane glycoprotein that is normally expressed by neural stem cells, endothelial precursor cells, and hematopoietic stem cells [60–62] and has become a distinctive marker of GSCs. CD133 levels are highly correlated with cells' clonogenicity, as shown by* in vitro *models; this has led some to hypothesize that glioblastomas are derived from CD133^+^ cells, but it is well known that some glioblastomas are CD133^−^ [5, 6, 20, 63–65]. Some studies have shown that these cells do not differ in gene expression or long-term survival rates and that they may even coexist in glioblastomas [66, 67]. High levels of CD133^+^ have been associated with progression and survival (independently of tumor grade, the extent of resection, or the patient's age) as well as with tumor regrowth and a high risk of dissemination. In CD133^−^ cells, on the other hand, investigators have been able to use CD15 as a GSCs marker [68–70].

In recent studies, it was shown that glioblastomas can exhibit different phenotypes and cell clones with distinct tumorigenic potential. In other words, the heterogeneity of tumors may be responsible for therapy resistance, migratory pattern, tumor invasion, proliferation, chemoresistance, tumor maintenance, self-renewal characteristics, tumor initiation, and oncogenic potential. Several studies have identified CD44, CD155, EGFR, L1CAM, A2B5, and integrin A6 as being responsible for the development of these characteristics. This highlights the need for studies that can identify distinct patterns of superficial markers that will distinguish GSCs to an efficient target therapy [8–12, 32–34, 71, 72].

Once the neurogenic niches house the NSCs (cells with a relatively large chance of becoming cancerous cells) and support the maintenance, survival and proliferation of these cells, they become the most vulnerable sites for growth and proliferation of transformed cells. Given that the SVZ is the largest neurogenic niche, it is believed that this region gives rise to the highest number of glioblastomas. However, GSCs and their progeny are not restricted to neurogenic niches; they can migrate away from their place of origin, as demonstrated by the presence of tumors in other brain regions.

**Figure 1:**
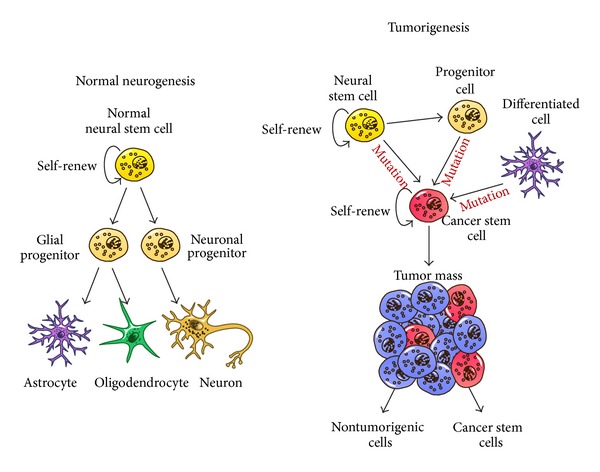
Cancer stem cell hypothesis. On the left, normal NSCs of the adult organism undergo extensive self-renewing division and give rise to a progenitor cell that differentiates into the three main neural lineages: neurons, astrocytes, and oligodendrocytes. On the right, CSCs are derived from physiological NSCs, progenitor cells, or mature brain cell, which acquire the ability to generate tumors following genetic mutations. The tumor mass is composed by different cell populations. Most of these cells appear to be nontumorigenic and only a small subpopulation of them represent the CSCs.

Despite the consistent body of evidence supporting NSCs as cells that give rise to gliomas, the possibility that these tumors arise from a fully differentiated cell type, such as a mature glial cell, has not been excluded [6, 73] (Figure 1). Astrocytoma mouse models have used combinations of oncogenic overexpression and/or tumor suppressor inactivation to induce tumor formation [74, 75], and some of these models have not been limited to NSCs.

To investigate the increased invasiveness of gliomas with Rictor mTORC2 signaling pathway overexpression, Bashir and colleagues [76] inserted human Rictor transgene strains into mice. This Rictor strain was crossed with mice expressing a recombinase limited to the glial compartment (astrocytes and oligodendrocytes) and resulted in the formation of multifocal intermediate and low-grade gliomas. In another recent study, transduced mature astrocytes with loss of p53 and oncogene overexpression simulated pivotal features of glioma pathogenesis [77]. These data obviously contradict the notion that gliogenesis arises solely from NSCs and adds fuel to the ongoing debate: is gliomagenesis a stem cell disorder or a reacquisition of stem cell characteristics?

## 4. Perivascular Niche

GSCs are found in a microenvironment that is very similar to that of normal stem cells. This microenvironment provides an ideal condition for tumor maintenance; however, it does not have the structural organization and stability generally associated with stem cell niches, and it also cannot be defined by a single location [78]. The tumor perivascular niche (PVN) is composed of a heterogenous group of cell types, including astrocytes, endothelial cells, macrophages, microglia, nontumor initiating cells, and brain tumor stem-like cells [79].

Tumors require a large amount of nutrients and oxygen to support their rapid growth, which occurs mostly during angiogenesis. This is often observed in cases of more aggressive brain tumors with large angiogenic activity, including endothelial hyperplasia and microvascular proliferation [80]. The vascular niches in brain tumors are abnormal and contribute directly to the generation of GSCs and tumor growth. Moreover, these niches protect the GSCs from environmental aggression and, in the process, provide resistance to conventional therapies [81]. Furthermore, there is a reciprocity between GSCs and their microenvironment: GSCs are capable of modulating their own microenvironment to produce signals to recruit other immature cells in the vicinity. One example is VEGFs secreted by GSCs, which are able to stimulate the growth of endothelial cells that support the local vascular environment [4, 82].

## 5. The Hypoxic Microenvironment

Hypoxia in the microenvironment is a characteristic of malignant tumors. In GBM patients, hypoxia is associated with tumor aggression and a negative prognosis [83]. Vascularization acts as a neoplastic feeding source and, due to the rapid tumor expansion, the vessels are often disorganized and unable to adequately deliver oxygen [84]. When the vasculature irrigates inefficiently, the low oxygen tension induces neovascularization in order to meet the tissue's needs [85, 86].

These cellular responses to hypoxia are commonly regulated by the transcription factor system of the hypoxia-inducible factors (HIFs) [87]. HIFs are heterodimers composed of an oxygen-sensitive HIF*α* subunit and a constitutively expressed HIF*β* subunit. Under normal oxygen conditions, HIF1*α* binds to the tumor suppressor protein von Hippel-Lindau (vHL); this interaction ubiquitinates and targets the HIF1*α* to the proteasome, where it is degraded. Under conditions of hypoxia, however, the interaction between HIF*α* and vHL is abrogated; as a consequence, HIF*α* becomes stabilized, leading to dimerization. It then binds to hypoxia-responsive elements (HREs) on the promoters of target genes that are often involved in modulating cell survival, motility, and metabolism [88, 89]. The activation of HIF*α* also plays a regulatory role in the expression of VEGF and inducible nitric oxide synthase (iNOS), facilitating angiogenesis and the tumor cell's access to the circulatory system [90]. Two HIF*α* subunits, HIF-1*α* and HIF-2*α*, are primarily responsible for regulating the tumor's adaptation to hypoxia. HIF-1*α* and HIF-2*α* are structurally similar in their DNA binding and dimerization domains; however, they can play nonoverlapping roles in tumor progression due to their unique target genes and different oxygen requirements for activation [85, 89, 91].

HIF-1*α* is widely expressed in several tissues, including normal neural progenitors, and is able to regulate cancer stem cell proliferation and survival. On the other hand, HIF-2*α* shows a more restricted expression pattern and is associated with cancer initiation or tumor progression, making it an attractive therapeutic target [89]. Interestingly, it has been shown that HIF-2*α* is able to promote a more stem-like phenotype in nonstem cancer cells, upregulating some key stem cell factors such as Oct4, Nanog, and c-Myc [92].

Several studies have demonstrated the importance of hypoxia and HIF in tumor biology and in the maintenance of GSCs, as well as their role in chemotherapy and radiotherapy resistance. Despite progress in recent years, a better understanding of this process is still needed for the development of new therapeutic strategies.

## 6. GSC Signaling Pathways

Signaling pathways can play a crucial role in the biology of physiological stem cells. When several of these pathways are dysregulated, they can lead to tumor initiation, progression, and metastasis. Some examples of these are Notch, bone morphogenetic protein (BMP), Wnt/*β*-catenin, sonic hedgehog (Shh), and STAT3.

Notch receptors are involved in several biological functions, including cell proliferation, differentiation, survival, and tumorigenesis [13]. Signaling by the Notch receptor occurs via cell-cell contact. Four Notch genes (Notch 1 to 4) have been identified in mammals, which act as transmembrane receptors for the Jagged (Jag1-2) and Delta-like (Dll1, 3, 4) ligands. When the pathway is activated, the receptor is cleaved and its intracellular region is translocated to the nucleus, acting as a transcription factor in conjunction with the CBF-1 (C promoter binding factor-1) protein. This is followed by the expression of transcriptional repressor genes such as Hes1 and Hes5, which repress the expression of proneural genes, thereby inhibiting neuronal differentiation. Thus, when activated, Notch signaling leads to the maintenance of the NSC population, while its inactivation induces neuronal differentiation [46]. It has been reported that Notch signaling is upregulated in GSCs, leading to uncontrolled self-renewal patterns [56, 93]. Moreover, Notch pathways have been shown to promote therapy resistance. Blocking Notch pathways depletes CD133-positive glioblastoma cells, thus decreasing tumor sphere formation, GCS proliferation, and xenograft growth and increasing differentiation [21].

In parallel, BMPs are a family of cytokines that regulate the proliferation, apoptosis, and differentiation of NSCs; this signaling process is a potent inhibitor of neurogenesis, blocking the production of neurons by inducing adult NPCs to adopt a glial fate [94]. The BMPs also act in GSCs, promoting astrocyte-like differentiation and inhibiting cellular proliferation [30]. BMP4 inhibits GSC proliferation via the downregulation of cyclin D1 and induces apoptosis by inducing Bax expression and inhibiting Bcl-2 and Bcl-xL [95]. Experimental studies have shown that the treatment of cultured GSCs with BMPs reduces the size of the tumors grafted into mice and prolongs the animals' survival [96].

Another candidate pathway able to regulate neuronal differentiation of adult NSCs and modulate GSC self-renewal is the Wnt/*β*-catenin signaling pathway [38]. In the Wnt pathway, the signal is transmitted from the surface to the nucleus through the *β*-catenin protein. In the absence of signal, a complex of proteins containing glycogen synthase kinase 3*β* (GSK3*β*) phosphorylates the cytoplasmic *β*-catenin, which is then degraded by proteasomes. When the Wnt signal is activated, the activity of GSK3*β* is inhibited, resulting in the accumulation of *β*-catenin. The accumulated *β*-catenin translocates to the nucleus and induces the expression of growth-related genes [97, 98]. Alterations in the Wnt pathway of glioblastomas lead to a negative prognosis. A selective inhibition of the Wnt signaling pathway in GSCs decreases cell proliferation, migration, and chemoresistance [22].

Other lines of evidence suggest that an altered Shh signaling pathway (generally associated with adult neurogenesis [48]) may lead to different types of cancer (solid and nonsolid) and is also associated with tumor development, proliferation, tumorigenesis, and metastasis [14, 15]. Shh is an important morphogen that is secreted at various stages of development. The binding of Shh to its receptor Ptch (patched) relieves Smo (Smoothened) inhibition, which in turn leads to the transcription of proteins from the Gli family (transcription factor). This Shh/Gli signaling pathway is necessary for CSC proliferation, self-renewal, and survival [14, 15]. Treatment of GSCs-derived neurospheres with the Hedgehog inhibitor cyclopamine inhibits CSC proliferation and self-renewal [99].

Finally, STAT3 (a member of the STAT family of cytoplasmic transcription factors) has been implicated in NSC development [100] and also in the formation of many types of tumors, including GBM [101]. STAT3 is activated by many cytokine and growth factor receptors. When activated, STAT3 enters the nucleus and triggers the gene expression of many procancerous proteins associated with cell cycle progression, antiapoptosis, angiogenesis, migration, and invasion [31]. Treating GSCs with small molecules that inhibit STAT3 DNA-binding has been shown to inhibit cell proliferation and the formation of new neurospheres from single cells [23]. Moreover, the inhibition of STAT3 also decreases the expressions of CD133 and c-Myc in GSCs and leads to apoptotic cell death [102].

## 7. Transcription Factors

Just like the signaling pathways, transcription factors play an important role in the maintenance and regulation of tumor cells. These factors are directly involved in the survival, maintenance, proliferation, and self-renewal of GSCs. Investigators have indicated that the transcription factors that play a significant role in brain tumors include Bmi1, Olig2, c-Myc, Sox2, Oct4, and Nanog.

Authors agree that some transcription factors play an important role in inducing tumor cells to act like stem cells. This suggests that even a small error during neurogenesis can initiate a cascade of reactions that may result in the formation of a glioblastoma.

Belonging to the family of Polycomb group proteins (which play the role of epigenetic regulators during the embryonic period), the Bmi1 is a component of the Polycomb Repressive Complex 1 (PRC1) found in undifferentiated neural stem cells. The PRC1 supports the maintenance of neural stem cell function and contains tumor-suppressor mechanisms. When cancer cells silence these mechanisms, there is a reduction in the amount of normal neural stem cells and a delay in the process of gliogenesis [103]. A significant link has been found between the manifestation of an aggressive phenotype of glioblastomas and high levels of Bmi1, as this seems to activate the nuclear factor kappaB (NF-kappaB). This factor is also activated in several other cancers and results in the increased regulation and activation of matrix metalloproteinase-9 (MMP-9), which is responsible for the destruction of extracellular matrix and basal membranes [104]. However, some studies suggest that such high values of Bmi1 in several tumors are the result of other mutations: when tested in* in vivo* transgenic mice models (compared to* in vitro* models), Bmi1 was observed to have a low proliferative effect, a low effect on fetal and adult neurogenesis, and a low effect on glial differentiation. Furthermore, it did not result in an increased capacity for self-renewal and neurogenic potential [105].

Recent studies have demonstrated that gene silencing of Bmi1, for example, by MicroRNA-218 (miR-218), MicroRNA-128 (miR-128), or epigenetic regulation of Survivin, results in decreased rates of tumor cell invasion, migration, proliferation, and self-renewal. Furthermore, the absence of these factors leads to gliogenesis, and some of these mechanisms are essential for normal and neoplastic cells to survive following Bmi1-induced proliferation [24–26].

Olig2 plays an important role in CNS development during the embryonic phase as well as in malignant glioblastomas during adulthood (for a detailed review, see [106]). Olig2's triple serine phosphorylation regulates the suppressive action of p53, which triggers proliferation in normal and malignant neural progenitors. However, this state of phosphorylation does not seem related to the specification and terminal differentiation of oligodendrocytes [107, 108]. Some possible transcripts involved in the promotion of quiescence and the differentiation state in Olig2 tumor cells seem to be deleted during tumorigenesis. Glioblastoma cells share characteristics with oligodendroglial progenitor cells, such as the fact that tumorigenesis is initiated by a glial progenitor-like cell [109]. Appolloni et al. showed that when Olig2 is silenced (or when this effect is mimicked by high levels of other factors, e.g., Pax6 or ID4), tumorigenesis and tumor growth are considerably reduced [28].

c-Myc, Oct4, and Sox2 (alongside Klf4) are used to reprogram embryonic and adult cells to induce pluripotency [16]. These factors are also associated with high-grade glioblastomas, promoting tumorigenic activity, glioma stem cell self-renewal, neurosphere formation, glioma stem cell proliferation, and in some cases—like c-Myc—acting as a GSC-specific survival factor [17–19, 27, 29, 110–113]. Glioma stem cells express high levels of c-Myc, and their proliferation and cell cycle progression are also regulated by c-Myc (see Table 1). The loss of this oncogenic factor induces GSC apoptosis and reduces neurosphere formation, while the knockdown of c-Myc inhibits GSCs' tumorigenic potential [29]. In a recent study, Elsir et al. studied the correlation between Nanog, c-Myc, Oct4, Sox2, and Klf4 in high-grade glioblastomas, low-grade glioblastomas, and low-grade astrocytomas. They observed the expressions of Oct4, Sox2, and Nanog in more than 50% of tumor cells and showed a possible correlation between these proteins in the regulation of the pluripotency and self-renewal of GSCs. The main finding in this work was a possible regulatory pathway of these proteins in glioblastomas. This makes them safe biomarkers for future clinical approaches and deems Nanog a determining factor in the clinical outcome [114].

As described above, many transcription factors seem to be involved in the stem cell-like state of tumor cells. It is likely that the combined effects of these transcription factors are the main reason why it is so difficult to establish a promising treatment. Exactly how these factors promote tumorigenesis is yet to be clarified, but recent findings have shed a light on our understanding of the mechanisms underlying tumor cells.

## 8. Radioresistance and Chemoresistance

There are several hypotheses regarding the mechanisms of radio and chemoresistance. In terms of radioresistance, the influence of different signaling pathways seems to give GSCs the ability to repair DNA more rapidly and efficiently than normal cells. Polycomb group proteins (e.g., Bmi1) also influence DNA repair and when they are deficient, GSCs are sensitized to radiation. The autophagy system, the notch pathway, the Akt signaling, and Wnt proteins all seem to contribute to the resistance of GSCs to radiotherapy, and some of these mechanisms affect both tumor cells and normal stem cells. In terms of chemoresistance, some theories implicate ABC drug transporters, which are regulated by Akt and are responsible for activating the efflux of various substrates across extra- and intracellular membranes; the participation of CD133 cell markers and the notch and shh signaling pathways that interact with DNA repair machinery have also been implicated. For a thorough review of this issue, see [115].

More studies need to be conducted to better understand the specific mechanisms underlying drug and radiation resistance, as well as how these mechanisms operate to make GSCs resistant to these clinical approaches. One great challenge to establishing a target therapy is that various mechanisms involved in brain tumors are basically the same mechanisms recruited in neurogenesis, which raises the following questions: how far can we go with an efficient target therapy without compromising normal cells? How can we eliminate a tumor without eliminating the normal stem cells that are necessary for recovering damaged areas? Clearly, there is a great need for studies that can identify the heterogeneous phenotype in GSCs in order to identify efficient target therapies.

## 9. Conclusion

Glioblastoma multiforme is one of the most aggressive forms of brain tumor and is associated with poor outcome and low survival rates. Despite all the current available treatments, surgery continues to be the most efficient option, although it has not been associated with high rates of improvement. Recent studies have focused on the main factors that initiate gliogenesis. Several hypotheses aim to describe the mechanisms involved in a normal cell's transformation into a malignant cell. Problems with signaling pathways or transcription factors—as well as other minor errors that may occur during neurogenesis—have been shown to guide neural stem cells toward a malignant phenotype. However, the greatest difficulty lies in the fact that these mechanisms are shared between normal cells and tumor cells.

These shared mechanisms are highly important for normal cell growth, proliferation, self-renewal, and differentiation, but they are also important for tumor cell survival and proliferation. Knowledge about malignant tumors allows us to better understand the behavior of malignant cells and to unveil the mechanisms that initiate tumorigenesis. This would represent an important starting point towards winning the battle against cancer.

## Figures and Tables

**Table 1:** Main mechanisms involved with GSCs.

Glioma stem cells			
Main features	Surface markers	Signaling pathways	Transcription factors
Tumorigenesis	L1CAM [8], EGFR [9], IntegrinA6 [10], CD155 [11], A2B5 [12]	Notch [13], Shh [14, 15]	Olig2 [16], Oct4 [17], Sox2 [17], Nanog [18, 19]
Self-renewal/proliferation	EGFR [9], CD133 [20], IntegrinA6 [10]	Notch [21], Shh [14, 15], Wnt [22], STAT3 [23]	Bmi1 [24–26], Sox2 [27], Nanog [18, 19], Olig2 [28], c-Myc [29]
Differentiation	—	BMP [30]	—
Survival	—	Shh [14, 15], STAT3 [31]	Bmi1 [24–26], c-Myc [29]
Migratory pattern/metastasis	CD44 [32], CD155 [11]	Wnt [22], Shh [14, 15], STAT3 [31]	Bmi1 [24–26]
Tumor invasion	CD44 [33], CD155 [11]	STAT3 [31]	Bmi1 [24–26]
Therapy resistance	L1CAM [34]	Notch [21], Wnt [22]	—

This table lists the markers, signaling pathways, and transcription factors related to specific features of GSCs.
